# Histological Changes in Adipose Tissue: An Alarm When Methamphetamine Is Targeted for Weight Loss Purposes 

**DOI:** 10.29252/wjps.10.1.53

**Published:** 2021-01

**Authors:** Fariba Jaafari-Sheybani, Seyed-Ebrahim Hoseini, Davood Mehrabani, Amin Derakhshanfar, Feridoun Karimi-Busheri

**Affiliations:** 1 Department of Biology, Shiraz Branch, Islamic Azad University, Shiraz, Iran;; 2 Stem Cell Technology Research Center, Shiraz University of Medical Sciences, Shiraz, Iran;; 3 Burn and Wound Healing Research Center, Shiraz University of Medical Sciences, Shiraz, Iran;; 4 Center of Comparative and Experimental Medicine, Shiraz University of Medical Sciences, Shiraz, Iran;; 5 Department of Oncology, University of Alberta, Edmonton, Alberta, Canada;; 6 Diagnostic Laboratory Sciences and Technology Research Center, School of Paramedical Sciences, Shiraz University of Medical Sciences, Shiraz, Iran.

**Keywords:** Methamphetamine, Behaviour, Anxiety, Adipose, Rat

## Abstract

**BACKGROUND:**

Methamphetamine (METH) may be administered for weight loss purposes and to understand the METH side-effects more in details, this study aimed at determining the effect of METH on changes in adipose tissue in experimental rats.

**METHODS:**

Forty five male Wistar rats were randomly allocated to three equal groups. Group 1 was experimental receiving METH [0.4 mg/kg, subcutaneously (S/C), 0.6 mL/rat] for 3 weeks, group 2 was the sham group receiving normal saline (0.6 mL/rat, S/C) and the 3^rd^ group was the control receiving distilled water, identically. The elevated plus maze test was used to confirm cognitive impairment and distraction as anxiety and to verify addiction to METH by assessing the percent time spent in open arm (OAT), the percent time spent in closed arm (CAT), the percent time spent in central parts and head dipping over the side of the maze. Adipose tissue was assessed histologically 7, 14 and 21-days after interventions.

**RESULTS:**

A significant increase in anxiety level, and histologically inflammation, degeneration and necrosis in adipose tissue were visible after METH use.

**CONCLUSION:**

METH use resulted in a significant inflammation and necrosis in adipose tissue denoting to the dangers of METH use, when recreationally targeted for weight loss purposes.

## INTRODUCTION

A prominent characteristic of drug addiction is the persistent effort to look for and to acquire a drug, at the expense of other healthy behaviors and negative consequences.^1^ Among drug addictions, d-Methamphetamine Hcl (METH) addiction is a prevalent health concern all over the world without any confirmed pharmacological treatment.^2^ World Health Organization’s (WHO) report shows rate of mortality up to 70 to 80% in developed and 40 to 50% in developing countries due to diseases and drug abuse.^3^

Based on United Nations’ Office on Drugs and Crime report in 2013, Iran has the fifth rank in terms of for MTH use^4^ with a rate of 18.5% among 15- to 25-year-old individuals in Tehran, Iran and 3.4% among students of Birjand and Gilan in northern Iran.^5^ United Nations Office on Drugs and Crime has reported a global use of 0.3-1.1% for amphetamine-type stimulants (13.8-53.8 millions).^6^ In US, METH use ranks a significant societal and economic burden, costing 23 billion dollars per year.^7^ METH is an amphetamine type stimulant that can lead to a drug desire, cognitive impairment and distraction.^8^ It was shown that glutamatergic, serotoninergic and dopaminergic systems in the brain are changed with co-exposure to drugs of abuse such as METH.^9^ METH is considered among the highly potent and severely addictive psychostimulants with a relapse manifested after a long period of abstinence.^10^ Although several users would like to quit METH use, the majority of them cannot afford, because of the lack of efficacious treatment measures.^6^

The majority of METH-dependent users who search for the drug treatment would return to METH use again within 6 months.^11^ Females were shown to be more sensitive to METH with more reinstatement behaviour.^12^ METH was demonstrated to cause a potentially lethal increase in core body temperature, or hyperthermia,^13^ while METH-induced hyperthermia has a prominent role in the thermogenic brown adipose tissue that can lead to weight loss.^14^ In animals, the physiological effects of METH use were studied revealing a rise in heart rate, arterial pressure, body temperature and locomotor activity.^14^ METH may be administered for weight loss purposes and to understand the METH side-effects more in details, this study aimed at determining the effect of METH on changes in adipose tissue in experimental rats.

## MATERIALS AND METHODS

METH (Sigma-Aldrich, USA) was provided from Shiraz Branch of Islamic Azad University, Shiraz, Iran for research purposes. Before being used in all experiments, it was dissolved in normal saline. Forty five male Wistar rats (8-week old, 200-220 g) were purchased from Comparative and Experimental Medicine Center, Shiraz University of Medical Sciences, Shiraz, Iran. All rats were kept in standard cages in groups of 2-5 at 22 °C, 70% humidity, and 12-h light/dark cycle (lights on at 8:00 a.m.), while they had free access to a standard diet and tap water, *ad libitum*. Before interventions, they were adapted to their condition. The study protocol was approved by Ethics Committee of Islamic Azad University, Shiraz Branch (7-E-IR-MIAU.REC.80-B-2018) based on Helsinki Declaration and Iran Veterinary Organization guidelines of working with animals. Thirty rats were randomly allocated to three equal groups of 10 animals. The first group was experimental receiving METH (0.4 mg/kg), subcutaneously in 0.6 mL volume for 3 weeks as described before.^12^ The second group was sham receiving normal saline (0.6 mL, subcutaneously) for 21 days and the third one was the control group receiving distilled water, identically for 3 weeks. Daily experiments were done in the morning from 08:00 to 12:00 AM. 

The plus-maze test was used to determine the anxiety level and to verify addiction to METH with two opposite open arms (50×10 cm), while the two opposite arms were confined by walls of 40 cm height. The arms were connected by a central square (10×10 cm) as a “plus” shape elevated (50 cm) from the floor, while lit by dim light. All steps of interventions were recorded by a video-camera being above at 50° angle to the maze to record the determined scores of the animals’ anxiety assessment in an adjacent room. Quantification of the entries together with the percent time spent in open arm (OAT), closed arm (CAT), and central parts of the maze were recorded too. Head dipping was assessed to investigate the exploratory movement of head/shoulders of the rats over the side of the maze. OAT was regarded as a negative correlation with anxiety level, while CAT illustrated a positive correlation with anxiety level as mentioned before.^15^

When all 4 paws entered the arm, it was considered as an arm entry; and exit from an arm was regarded after the forepaws left that arm. All rats on test day were placed in the dimly illuminated laboratory undisturbed for at least 1 h before starting the experimentation. As rats are animals that are usually reactive to direct handling of man, a cylindrical cardboard tube was designed for individual transportation of them from the home cage to the plus-maze. Scoring was 5 min performed by an observer who was blind to the tests and the results were directly entered a PC computer. All tests were between 8.00 and 12.00 a.m. and the maze was thoroughly cleaned (wet and dry cloths) between successive experiments.

Animals were sacrificed after 7, 14 and 21 days following interventions. Adipose tissue was removed from abdominal and pelvic cavity and left in 10% buffered formaldehyde for one week. The dehydration was done after two changes in cold ethanol, clearing after three changes in cold xylene, and embedding after placing in paraffin at 53°C. Tissue section was further prepared serially at 5-µm thickness, left at 37°C for an hour to be dried and finally stained by haematoxylin and eosin. The prepared sections were visualized using a light microscope. The obtained data were exhibited as mean±SEM and statistically analyzed using SPSS software (Version 21, Chicago, IL, USA) by one-way ANOVA, Post-hoc Tukey’s and independent t tests. P values≤0.05 were defined statistically significant.

## Results

**Fig. 1:**
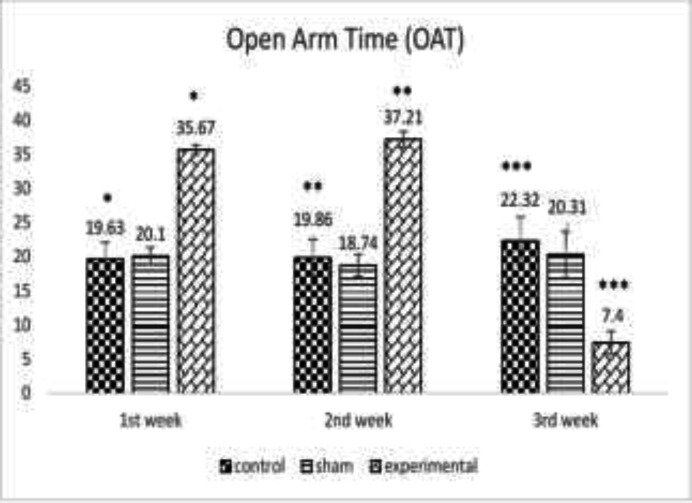
The effect of METH on the mean percentage of open arm time (OAT) at the end of the first, second and third week between the control, sham and the experimental group *(*p*<0.05), **(*p*<0.0005)

**Fig. 2:**
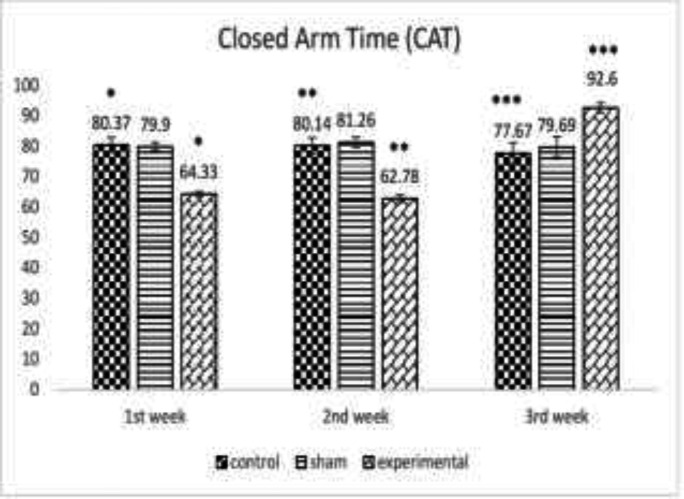
The effect of METH on the mean percentages of time spent in the closed arm time (CAT) at the end of the 1^st^, 2^nd^, and 3^rd^ week between the control, the sham and the experimental group *(*p*<0.05), **(*p*<0.0005)

The effect of METH on OAT at the end of the first, second and third week following METH use showed a significant difference between the groups (*p*<0.05, *p*<0.0005, respectively) revealing an increase in anxiety level following METH use until 3 weeks (Fig. 1). Figure 2 denotes to the effect of METH on CAT until three weeks illustrating a significant difference between the groups (*p*<0.05, *p*<0.0005, respectively) demonstrating a significant increase in anxiety level following METH use. 

**Fig. 3:**
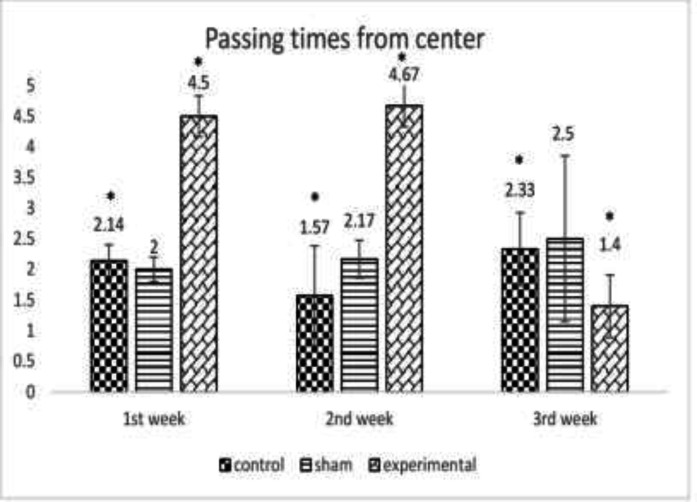
The effect of METH on the mean number of times rats passing from the center at the end of the first, second and third week between the control, the sham and the experimental group *(*p*<0.01)

**Fig. 4:**
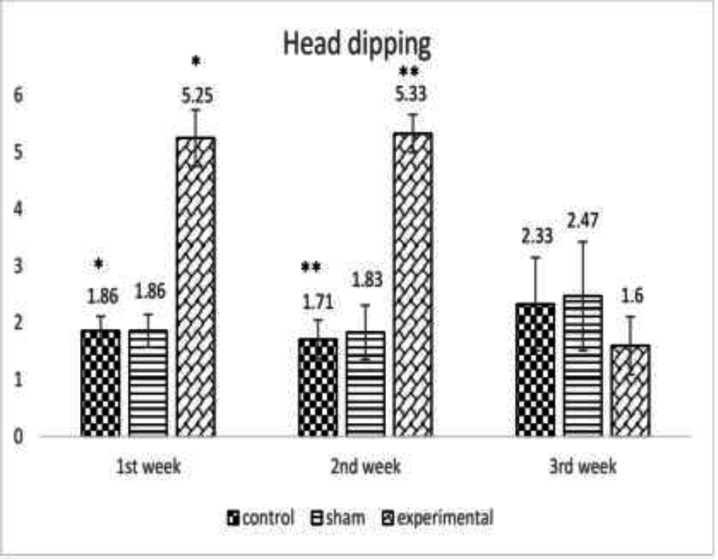
The effect of METH on the mean number of peeks at the end of the first, second and third week between the control, the sham and the experimental group *(*p*<0.005), **(*p*<0.005)

**Fig. 5:**
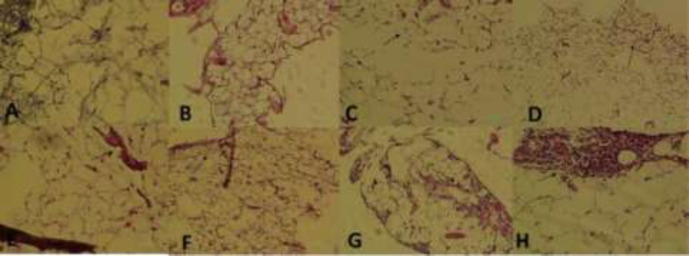
A photomicrograph of a paraffin section of the adipose tissue following METH use in three groups of experimental, sham and control rats. **A:** Control, normal (X200, H & E).; **B:** Sham, 1^st^ week, normal (×100, H & E); **C:** Sham, 2^nd^ week, disorganization (×100, H & E); **D:** Sham, 3^rd^ week, collapse (×100, H & E); **E:** METH, 1^st^ week, saponification, degeneration, necrosis (X200, H & E); **F:** METH, 2^nd^ week, saponification, degeneration, necrosis (X100, H & E); **G:** METH, 3^rd^ week, MNCs inflammation, steatites, necrosis (×100, H & E); **H:** METH, 3^rd^ week, MNCs inflammation, steatites, necrosis (×200, H & E)

When the effect of METH on percent time spent in central parts of the maze until the third week was investigated, a significant difference was observed between the groups (*p*<0.01) denoting to a significant increase in the anxiety level after METH consumption (Figure 3). Figure 4 presents the effect of METH on the mean number of head dipping after the first, second and third week of METH use confirming a significant difference between the groups (*p*<0.005, *p*<0.005, respectively) displaying a significant increase in anxiety level after METH administration. METH administration resulted in severe histological changes in adipose tissue including saponification, degeneration, infiltration of mononuclear cells, inflammation, steatites, and necrosis (Figure 5).

## DISCUSSION

METH is still illegally used for different purposes such as alertness, enhancement of sexual pleasure, and because of its availability, and cost-effective price is popular. Based on its anorectic effects and especially for weight loss purposes, it also abused among women, even during pregnancy that denotes to the public health importance of METH use.^14^^,^^16^ Its use is accompanied by several physiological alterations and health concerns.^17^ The experiments in conscious animals following METH use revealed increase in body temperature, locomotor activity, arterial pressure, and heart rate.^18^

It was shown that a single dose of METH can significantly influence basic homeostatic systems and protective functions.^19^ We showed degenerative changes in adipose tissue following METH use together with inflammation, and necrosis. Jamshidi *et al.* showed an increase in apoptosis and a decrease in growth kinetics of human adipose-drived mesenchymal stem cells following cannabis use.^20^^,^^21^ Our findings on inflammatory responses, degeneration and necrosis in adipose tissue following METH use are in agreement with these studies. Sazmand *et al.* (2018) have also investigated the effect of cannabis on growth of bone marrow mesenchymal stem cells in rat and demonstrated an increase in apoptosis and a decrease in proliferation of stem cells identical to our results after the substance use.^22^

Severe tissue lesions in rats following administration of cannabis have mentioned its use with caution.^23^ Histological findings after treatment with cannabis in rats denoted to inflammation, degeneration and necrosis in adipose tissue too confirmed with increased anxiety.^24^ Regarding METH, it was shown that METH can induce significant uterine pathology and anxiety in female Wistar rats confirming our findings in adipose tissue.^25^ The presence of METH in post-mortem adipose tissue due to its ante-mortem deposition was shown before.^26^ Many researchers have also studied on the effect of METH on core temperature indicating an increase in brown adipose tissue temperature (iBAT) in rat that can induce weight losses too.^27^ METH use was shown to increase the availability of biogenic amines directly at brown adipocytes by noradrenaline that was independent of physical exertion that can explain our findings too.^14^

METH abuse has prominent CNS side effects such as psychosis and depression.^28^^,^^29^ Alavijeh *et al. *(2019) showed that METH consumption induced anxiety-like behaviors via modulation of oxytocin receptors in methamphetamine addicted rats the same as our finding.^30^ These behavioral changes can also be due to the high and quick distribution of METH in the body and enhancement of synaptic and extra-synaptic levels of serotonin, noradrenaline and dopamine.^31^

## CONCLUSION

As we did not yet understand the full ranges and degrees of histological changes in the adipose tissue following METH administration that would be of great importance. We found that following an increase in anxiety-like behaviors after METH use, severe tissue injuries in the adipose tissue including inflammation, degeneration, and necrosis happened. These results can be added to the literature on public health importance and social concerns of METH use, especially when METH consumers target it recreationally for weight loss purposes. 
